# Association of historic redlining and present-day health in Baltimore

**DOI:** 10.1371/journal.pone.0261028

**Published:** 2022-01-19

**Authors:** Shuo Jim Huang, Neil Jay Sehgal

**Affiliations:** Department of Health Policy and Management, University of Maryland School of Public Health, College Park, Maryland, United States of America; Montclair State University, UNITED STATES

## Abstract

**Background:**

In the 1930s, the Home Owners’ Loan Corporation categorized neighborhoods by investment grade along racially discriminatory lines, a process known as redlining. Although other authors have found associations between Home Owners’ Loan Corporation categories and current impacts on racial segregation, analysis of current health impacts rarely use these maps.

**Objective:**

To study whether historical redlining in Baltimore is associated with health impacts today.

**Approach:**

Fifty-four present-day planning board-defined community statistical areas are assigned historical Home Owners’ Loan Corporation categories by area predominance. Categories are red (“hazardous”), yellow (”definitely declining”) with blue/green (“still desirable”/”best”) as the reference category. Community statistical area life expectancy is regressed against Home Owners’ Loan Corporation category, controlling for median household income and proportion of African American residents.

**Conclusion:**

Red categorization is associated with 4.01 year reduction (95% CI: 1.47, 6.55) and yellow categorization is associated with 5.36 year reduction (95% CI: 3.02, 7.69) in community statistical area life expectancy at baseline. When controlling for median household income and proportion of African American residents, red is associated with 5.23 year reduction (95% CI: 3.49, 6.98) and yellow with 4.93 year reduction (95% CI: 3.22, 6.23). Results add support that historical redlining is associated with health today.

## Introduction

Race and its intersection with place matters when it comes to health disparities. Structural racism—the interplay of (sometimes unintentional) processes and structures that reinforce racial inequities [1]—has a large role in continued health disparities [2–5]. Place, geography, and the built environment are understudied dimensions of structural racism in terms of their impact on racial differences in health [6]. These continuing structural contributions to health disparities can often be traced to historically discriminatory policies and practices, such as redlining.

The impact of redlining is part of a broader national conversation about the impact of racially discriminatory policy and systems on disparate racial impacts, particularly differences between Black and white Americans. The 2014 Ta-Nehisi Coates article “The Case for Reparations” in *The Atlantic* [7] brought back to the fore a national conversation about and made an argument for reparative investment to correct inequities suffered by Black Americans. Part of Coates’ thesis was that redlining led to systematic racial discrimination in housing throughout the twentieth century, which still impacts intergenerational wealth and life outcomes in Black communities today.

Racially discriminatory local and federal policies and redlining’s role were also publicly discussed in the context and aftermath of police murders of unarmed Black civilians. After the late-summer 2014 murder of Michael Brown, Ferguson, MO underwent an uprising in protest at his death and the sense that historical and current local government policies—including redlining—were unjust and led to police suppression of the Black community [8]. In Freddie Gray’s murder in Baltimore in April of 2015 and subsequent uprising, another explicit tie-in was made with the city’s history of redlining [9–11]. A public link was being made between how redlining creates racial residential segregation [12,13], and particularly the hypersegregation of Black residents from white residents [14] that concentrated and exposed Black residents both to environmental hazards and overpolicing [15].

## Background and literature review

### What is redlining?

In the latter half of the 1930s, the US Federal government authorized the Home Owners’ Loan Corporation (HOLC) to draw “security” maps for investment purposes [16]. These maps were drawn with color codes to reflect areas that were more or less safe for investment. Four color codes were used: green, blue, yellow, and red. Green areas were labeled “A Best”, Blue areas “B Still Desirable”, Yellow areas “C Definitely Declining”, and Red areas “D Hazardous”. Red signified the highest risk areas to HOLC, which led to the term “redlining”. These color-coded categories reflected race and were most likely drawn to discourage investment in majority Black neighborhoods [13]. Redlined areas were explicitly labeled as such due to a large proportion of Black residents, using language characterizing “detrimental influences” such as “infiltration of Negro” while yellow categories often included the tag “infiltration of Foreigners” [16–19].

### Evidence that redlining might still have impacts today

Even time-limited urban changes can have long-lasting cumulative effects. Michaels and Rauch [20] found that the differential collapse of Western Roman urbanization in Britain and France in the 6^th^ century CE differentially impacted the spatial efficiency of urbanization even 1500 years later in the 21^st^ century. Similarly, evidence supports that redlining still has cumulative impacts on various social factors today. Aaronson et al. [12] found that living in close proximity on two sides of differently graded borders—as represented in the 1930s HOLC security maps—is strongly associated nation-wide with increased residential racial segregation from the 1930s to today. The effect on residential racial segregation was particularly strong from 1930 to 1970 and the effect size began to decrease after 1970. Aaronson et al. provide additional support that maps were likely drawn with race in mind: only areas marked category D had a primarily Black population in the 1930s. In addition to increased segregation, Aaronson et al. found support for reductions in home ownership, house values, and credit scores throughout the 20^th^ century that are maintained even today nearly a century later. They also found evidence of “yellow-lining”: areas marked as category C also had disparate current outcomes when compared to higher rated areas. Using a different methodology, Appel and Nickerson [21] found that redlined neighborhoods had lower home prices in 1990 compared to surrounding areas, and that these discriminatory effects remained even after nearly 60 years. The presence of these discriminatory effects can be compounded across time: Massey et al. [22] found that Black residents of redlined neighborhoods face greater barriers to residential mobility than white residents that negatively impacts Black residents’ social and economic well-being.

### Potential impacts of redlining on health

A growing body of scholarship examines the relationship between neighborhood characteristics and health outcomes [23], though few studies have specifically considered the legacy of redlining. The shadows of historical redlining are seen on present day health where structural racism and neighborhood segregation persist. The potential pathways for redlining’s impact on health reflect those found in residential segregation [23] and fall across the entirety of the life course [9,23–40]. Proposed pathways span both differential exposures to environmental and social hazards (including both the proximity and imposition of hazards and the reduced presence of mitigatory systems such as reduced access to health services [23,27]) and possibly the concentration of populations that have previously suffered such hazards [25,29]. Lynch et al. [27] find that historical redlining was associated with sustained disinvestment and lending discrimination in Milwaukee neighborhoods, as well as poorer present day physical and mental health. Similarly, neighborhood quality is thought to affect environmental health and reduce access to resources that enable protective behaviors [28–31]. Kreiger et al. [24] found that residing in a previously redlined area in Massachusetts was associated with an elevated risk for late stage at cancer diagnosis, even for residents of census tracts with present-day economic and racial privilege.

Redlining’s impact on health through disinvestment potentially starts before birth and in childhood. O’Campo et al. [29] found that neighborhood risk factors were significant in predicting low-birth weight. Specifically, they found that low-income census tracts (those with less than $8000 per capita annual income) were associated with higher risk of low birthweight. In examining birth certificate data and HOLC grade, Krieger et al. [25] concluded that historical redlining may be a structural determinant of present-day risk of preterm birth in New York City. On the other hand, Mendez et al. [31] found that redlining as represented through current mortgage discrimination was not associated with pregnancy outcomes. However, Mendez et al. stated that there was not enough variation in their sample to estimate associations and hypothesized that redlining might have measurable health impacts in other realms.

Wilson et al. [28] show the pathway from historic redlining to worsened pollution and housing stock and ultimately to poor asthma outcomes. Similarly, Alexander and Currie [30] found that sustained racial segregation in neighborhoods in New Jersey is associated with increased prevalence of asthma in low birthweight children. Nardone et al. [26] report that historically redlined census tracts in California have significantly higher rates of emergency department visits due to asthma.

Neighborhood quality and redlining might have an impact on physical activity as well. Boone et al. [32] find that redlined neighborhoods in Baltimore had reduced availability of greenspaces. The availability of greenspaces, walkability, other neighborhood built environmental factors, and neighborhood income in Baltimore and Seattle were found to be significantly associated with physical activity and better physical quality of life [6], which are factors that are important for the prevention and management of chronic diseases [41].

Potentially negative health impacts can also be a manifestation of direct state subjugation of and lack of political responsiveness to marginalized populations through environmental injustice. McCoy [34] found that coal-fired plants were more likely to be sited in communities of color. Trangenstein et al. [33] find HOLC category best predicts slow responsiveness of liquor store closures to depopulation and store clustering in Baltimore. Pappoe [9] in examining Freddie Gray’s case in Baltimore, argues that government-enabled and sponsored housing segregation and concomitant impacts of underfunded education, overpolicing, and deliberate impoverishment is the best frame to understand both structural and interpersonal violence (including state-sponsored violence) against Black people living in Baltimore.

The totality of potential health impacts across the life course may be reflected in life-expectancy [25,40] and age-differentiated mortality. Richardson et al. find large differences in life expectancy by HOLC grade [40]. To our knowledge there has not been a study yet on associations with age-differentiated mortality.

### Methodological issues in measuring redlining

HOLC map categories do not map well onto other, more conventional geographical units. HOLC categories cross census tracts (both contemporaneous and current), as well as larger neighborhood boundaries. This non-cartographic categorization can cause issues in classifying neighborhoods according to HOLC category as well as obtaining representative sets of sociodemographic characteristics of neighborhoods in ecologic studies. Predominance, or the use of the largest category feature to assign a non-cartographic category to a conventional geographic unit, is commonly used across disciplines [42–46]. In studies where the unit of analysis is an individual or their address, these categorization issues are no longer relevant, but such access is almost always subject to restrictions. Studies under those restrictions for individual-level data have sometimes used multilevel models for area-level effects while using addresses only for precision in geographical unit assignment [24,25]. While smaller sizes of conventional geographical units, such as census tracts or block groups, may offer better resolution than larger units, such as CSAs, they are still susceptible to the ecological fallacy.

HOLC categories also do not necessarily function as “equal steps” away from each other [47]. Some studies have treated HOLC categories as intervals specifically for the purpose of examining potential determinants of those HOLC categories, but have also noted the limitations of taking such an approach [47]. Although changes in neighborhood demographic composition are by no means exogenous, HOLC maps cannot directly capture the demographic changes that have occurred over the last century nor other federal and local policies [12] that may either augment or reduce the potential demographic and health impacts of redlining in a given area.

## Objective

The objective of this paper is to examine whether redlining policies from nearly a century ago are associated with a lingering disparate impact on health outcomes at the community statistical area (CSA) level today. The specific case of Baltimore is examined both because of its place in the national conversation about redlining, and because of its availability of public data on both HOLC security maps and CSA health indicators including life expectancy, maternal and child health outcomes, and age-differentiated mortality (which has not been previously studied).

## Materials and methods

### Data sources and variables

CSA neighborhood maps were taken from the Baltimore Neighborhood Indicators Alliance (BNIA) [48]. This is a common set of CSA polygons that are each composed of several masked census tracts. These shapefiles include neighborhood characteristics such as median household income uptake and racial composition for each CSA in 2013. They also include various neighborhood health outcomes including 2013 life expectancy at birth (calculated using prevailing rates of death through the life span), teen pregnancy (per 1000 female residents aged 15–19), percentage of births at term, percentage of births at satisfactory birth weight, cases of elevated childhood lead levels per 1000 children 0–6, and mortality per 10,000 deaths in a five-year period at various age categories. Additionally, there are health process outcomes such as the uptake of prenatal care, as well as neighborhood features such as rate of liquor stores and fast-food restaurants. Shapefiles that include this data in a map were downloaded from BNIA.

The redlining map was taken from the Mapping Inequality [16] project on HOLC security maps. HOLC categories are assigned to areas within the current city of Baltimore geography and extend beyond the city of Baltimore’s boundaries. Given the age of the maps, not all of present-day Baltimore is categorized. Shapefiles that include this data in a map were downloaded from the University of Richmond’s Digital Scholarship Lab.

### Methods

#### Transformations and overlay

All mapping analysis was done in QGIS 2.18.13. BNIA’s CSA shapefiles were projected using WGS84, these were converted into WGS84 pseudo-Mercator projection to be compatible with the Mapping Inequality HOLC shapefiles so that intersect overlays could be performed.

In order to determine which map sections lined up with each other, the intersection was taken of HOLC categories and Baltimore CSAs. The intersection of two fields in two maps returns all sub areas.

In line with methods for determining the classification of minimum mapping areas [42,43], the largest HOLC category by area within a CSA was then taken to represent the CSA’s HOLC categorization. This method is used across several disciplines [44–46]. We do not follow Hillier’s [47] usage of interval HOLC categories: they were trying to find determinants of HOLC categories and had noted the problematic limitations with treating HOLC categories as interval rather than ordinal. Aaronson et al. [12] were looking specifically at boundary blocks and tracts, which is beyond the scope of this analysis.

### Statistical analysis

All statistical analysis was done in StataMP 16. The main independent variable of interest was HOLC category. Summary statistics were taken of HOLC category, life expectancy, median household income, and proportion of African American residents.

Next, our main dependent variable of life expectancy was regressed with ordinary least squares (OLS) against the main independent variable. Red and yellow lined CSAs were analyzed against a reference category of combined green/blue CSAs (for sample size reasons). A second OLS analysis was run with the control variable of median household income—to account for the independent non-HOLC effects of CSA income on health—and the control variable of proportion of African American residents—to account for other forms of anti-Black structural racism and racial composition change on health [2,29]. These measures cannot be fully disentangled from redlining, so we test for multicollinearity using variance inflation factor. We define our threshold of statistical significance to be *p* < 0.05.

Various other health outcomes across the life course were also regressed on HOLC category. These outcomes measures included mortality in BNIA-defined age bins (infancy, 1–14, 15–24, 25–44, 45–64, 65–84, and 85+), teen birthrate, percentage of births at term, percentage of babies born with satisfactory weight, and percentage of children testing positive for elevated blood lead levels. Process measures that were also regressed as dependent variables against HOLC category included percentage of mothers receiving prenatal care. Neighborhood factors that might impact health behaviors were regressed including the per population rate of liquor stores and fast-food restaurants in a CSA.

Finally, we ran a sensitivity analysis by redoing our first OLS analysis while excluding CSAs that have less than 25% HOLC coverage to see if our analysis is sensitive to CSAs that have low HOLC coverage.

## Results

### Mapping sample

**Fig 1:**
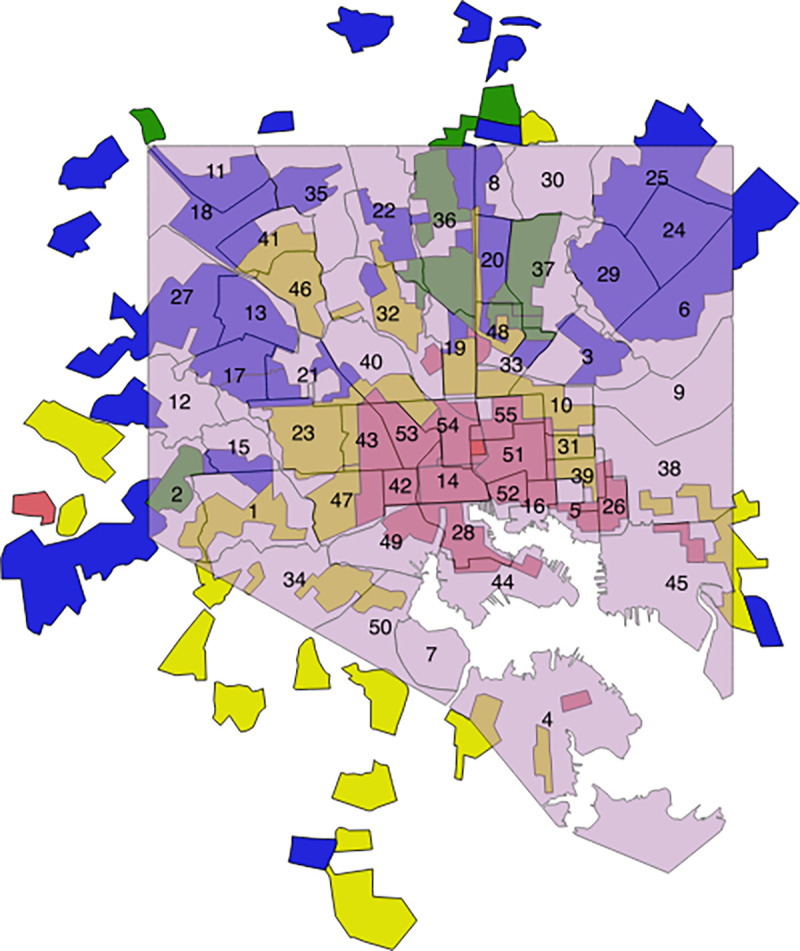
2013 Baltimore CSA neighborhoods (numbers refer to CSA names) in light purple overlaid on top of HOLC redlining map (red: “Hazardous”, yellow: “Declining”, blue: “Still desirable”, green: “Best”). HOLC redlining map republished from Nelson RK, Winling L, Marciano R, Connolly N, et al. Mapping Inequality. n.d. [cited 2017 Nov 6]. In: Nelson RK, Ayers EL, eds. American Panorama [Internet]. Richmond: University of Richmond Digital Scholarship Lab. Available from: https://dsl.richmond.edu/panorama/redlining/#loc=11/39.293/-76.808&city=baltimore-md under a CC BY license, with permission from University of Richmond Digital Scholarship Lab, original copyright 2016.

**Fig 2:**
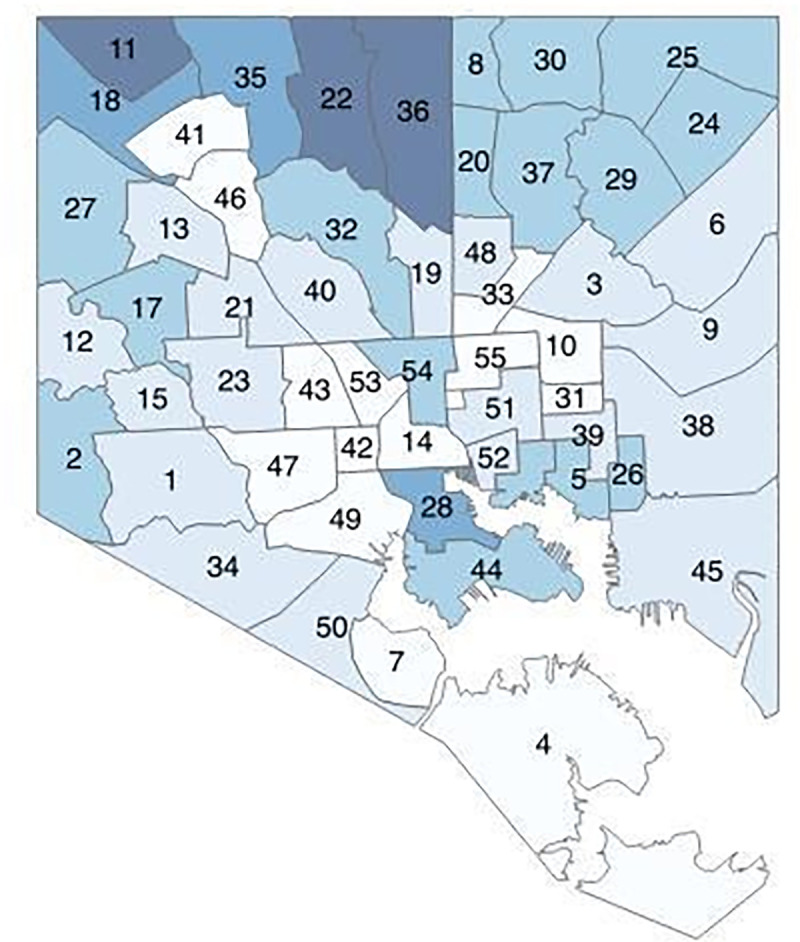
2013 Baltimore CSAs by life expectancy. Darker blue is longer life expectancy. Numbers refer to CSA names.

**Fig 3:**
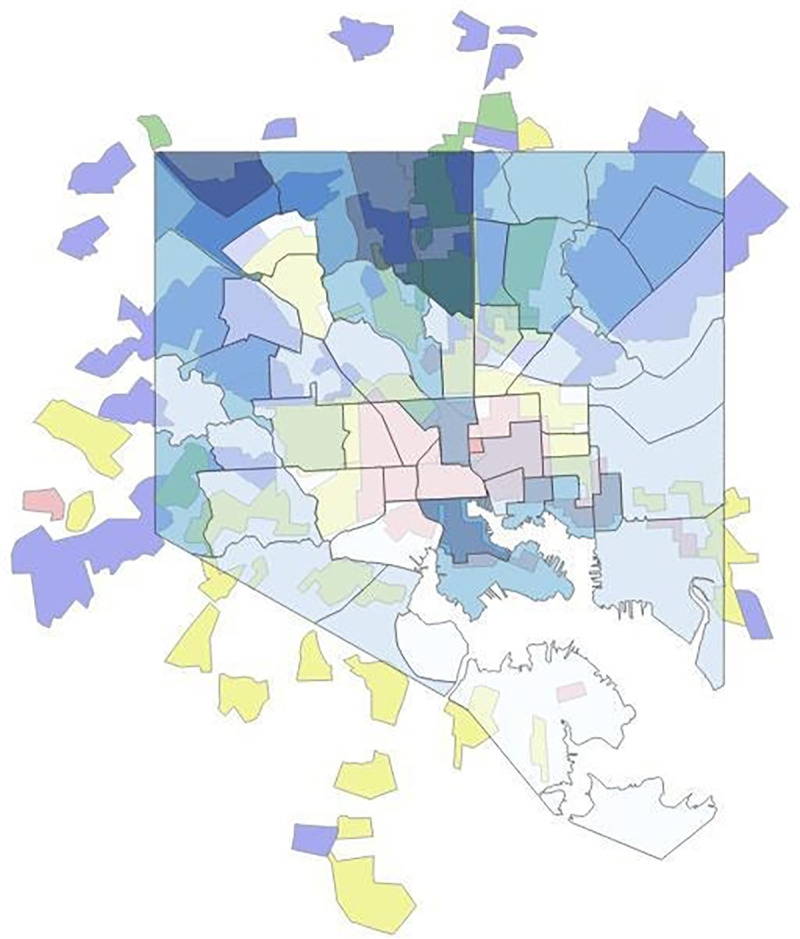
2013 Baltimore CSAs’ life expectancy overlaid on top of HOLC redlining map. HOLC redlining map republished from Nelson RK, Winling L, Marciano R, Connolly N, et al. Mapping Inequality. n.d. [cited 2017 Nov 6]. In: Nelson RK, Ayers EL, eds. American Panorama [Internet]. Richmond: University of Richmond Digital Scholarship Lab. Available from: https://dsl.richmond.edu/panorama/redlining/#loc=11/39.293/-76.808&city=baltimore-md under a CC BY license, with permission from University of Richmond Digital Scholarship Lab, original copyright 2016.

The BNIA shapefiles included 55 CSA polygons for Baltimore (Figs 1 and 2). The Mapping Inequality shapefiles for Baltimore included 42 HOLC categorized polygons (Fig 1). A total of 156 polygons were created with an intersect overlay between CSA polygons and HOLC polygons (Fig 3). One Baltimore CSA polygon (#7, Cherry Hill) was dropped from the analysis since there were no intersections with the Mapping Inequality shapefiles (the area was not assigned any HOLC categories in the 1930s). 54 primary polygons represented the remaining CSAs with HOLC categories assigned.

There were 14 CSAs that were classified as red using the predominant polygon by area. 19 were classified as yellow, 17 as blue, and 4 as green. Red and yellow polygons tended to be at the city core, while green and blue polygons tended to be at the northern peripheries (Fig 1).

### Summary statistics

CSA characteristics are fairly heterogeneous in Baltimore. The mean life expectancy at birth in the 54 CSAs was 73.3 years, with a standard deviation of 4.33 years, and ranging from a low of 66.0 to a high of 85.3. Mean of median CSA household income was $44,610 with a low of $13,887 to a high of $106,771. Mean African American proportion of the population was 61.2%, with a low of 2.44% to a high of 97.4% (Table 1).

**Table 1:** Summary statistics for 2013 Baltimore CSAs.

n = 54	Life Expectancy-years			Median HH Income-$			African American-%		
	Mean	Std Dev	Range	Mean	Std Dev	Range	Mean	Std Dev	Range
Total	73.3	4.33	66.0, 85.3	44610	20162	13877, 106771	61.2	33.2	2.44, 97.4
HOLC red (n = 14)	72.3	4.41	66.0, 78.8	47539	28853	13887, 92130	46.9	37.8	2.44, 96.4
HOLC yellow (n = 19)	70.9	2.32	66.8, 76.4	35482	9630	24175, 60104	62.5	31.8	10.7, 97.4
HOLC blue (n = 17)	75.9	4.16	71.2, 85.3	49430	19154	31701, 106771	70.1	29.1	6.86, 96.4
HOLC green (n = 4)	77.9	3.91	75.4, 83.8	57225	12220	47124, 74277	67.4	34.7	15.9, 90.0

### OLS regression

CSAs with red classification were statistically significantly associated at the 95% confidence level with a mean 4.01 years reduction (95% CI: 1.47, 6.55) in CSA life expectancy and CSAs with a yellow classification were associated with a mean 5.36 years reduction (95% CI: 3.02, 7.69) in CSA life expectancy (Table 2). Once CSA proportion of African Americans and CSA median household income were added to the model as controls, the effect was slightly stronger in red CSAs with a mean reduction of 5.23 years (95% CI: 3.49, 6.98) and slightly weaker in yellow CSAs with a mean reduction of 4.93 years (95% CI: 3.22, 6.63). This model accounted for 73% of the variability of the life expectancy response data around the mean. The Variance Inflation Factor of this model was 2.17, so multicollinearity seems less likely to be an issue. In the fully adjusted model, yellow classification is also significantly associated with mortality between the ages of 15–24 (mean 4.65 additional deaths per 10,0000 people), while yellow and red classifications are also significantly associated with mortality between the ages of 25 and 44 (mean 10.54 (yellow) and 6.19 (red) additional deaths per 10,000 people), mortality between the ages of 45 and 64 (mean 30.99 (yellow) and 36.4 (red) additional deaths per 10,000 people), mortality between the ages of 65 and 84 (mean 74.65 (yellow) and 95.47 (red) additional deaths per 10,000 people) (Table 3).

**Table 2:** Multivariable ordinary least squares analysis of Baltimore HOLC and 2013 life expectancy.

Independent variables: n = 54	Model 1: life expectancy-years		Model 2: life expectancy-years	
	Effect [95% CI]	P-value	Effect [95% CI]	P-value
HOLC Blue/Green	ref		ref	
HOLC Yellow	-5.36 [-7.69, -3.02] *	p<0.001	-4.93 [-6.63, -3.22] *	p<0.001
HOLC Red	-4.01 [-6.55, -1.47] *	0.003	-5.23 [-6.98, -3.49] *	p<0.001
Median HH Income-$1000			0.06 [0.01, 0.11] *	0.028
African American-%			-0.06 [-0.09, -0.03] *	p<0.001

* Significant at the 95% confidence level. Unit of analysis is the community statistical area. Dependent variables in columns, independent variables and covariates in rows. Mean and 95% CI reported. Model 1: Life expectancy regressed on HOLC yellow and HOLC red vs. HOLC blue/green. Model 2: Added neighborhood median household income and proportion African American as controls. Median household income and percent African American are controls and effects should not be interpreted independently.

**Table 3:** Multivariable ordinary least squares analysis of Baltimore HOLC and 2013 mortality by age bins.

HOLC Blue/Green reference	HOLC Yellow		HOLC Red		Median HH Income-$1000		African American-%	
Dependent variables: n = 54	Effect [95% CI]	P-value	Effect [95% CI]	P-value	Effect [95% CI]	P-value	Effect [95% CI]	P-value
mortality up to age 1 deaths in 5 years-per 10000	1.98 [-0.39, 4.35]	0.100	2.01 [-0.42, 4.44]	0.102	-0.01 [-0.08, 0.06]	0.872	0.11 [0.07, 0.15] *	**p<0.001**
mortality age 1–14 deaths in 5 years-per 10000	0.13 [-2.16, 2.43]	0.907	1.66 [-0.69, 4.01]	0.163	-0.05 [-0.11, 0.02]	0.177	-0.01 [-0.05, 0.03]	0.647
mortality age 15–24 deaths in 5 years-per 10000	4.65 [1.19, 8.1] *	**0.009**	3.51 [-0.02, 7.05]	0.051	-0.02 [-0.12, 0.09]	0.744	0.09 [0.03, 0.15] *	**0.004**
mortality age 25–44 deaths in 5 years-per 10000	10.54 [6.51, 14.57] *	**p<0.001**	6.19 [2.07, 10.32] *	**0.004**	-0.07 [-0.19, 0.05]	0.266	0.24 [0.17, 0.31] *	**p<0.001**
mortality age 45–64 deaths in 5 years-per 10000	30.99 [18.64, 43.35] *	**p<0.001**	36.4 [23.75, 49.06] *	**p<0.001**	-0.74 [-1.1, -0.37] *	**p<0.001**	0.19 [-0.03, 0.4]	0.09
mortality age 65–84 deaths in 5 years-per 10000	74.65 [32.22, 117.07] *	**0.001**	95.47 [52.03, 138.91] *	**p<0.001**	-0.8 [-2.05, 0.45]	0.205	0.4 [-0.34, 1.14]	0.284
mortality age 85 and up deaths in 5 years-per 10000	136.9 [-4.86, 278.65]	0.058	103.16 [-41.99, 248.31]	0.16	4.31 [0.13, 8.49] *	**0.044**	2.58 [0.1, 5.06] *	**0.042**

* Significant at the 95% confidence level. Unit of analysis is the community statistical area. Dependent variables in rows, independent variables and covariates in columns. Mean and 95% CI reported. BNIA pre-defined age bin mortality rates are individually regressed on HOLC yellow and HOLC red vs. blue/green, with covariates of neighborhood median household income, proportion of African American residents. Effects are reported as additional deaths in a 5 year period per 10000 residents within an age group for the relevant HOLC grade (yellow or red) as compared to the reference category of HOLC blue/green. Median household income and percent African American are controls and effects should not be interpreted independently.

HOLC classification was not significantly associated with infant mortality, mortality from ages 1–14, or mortality over the age of 85 (Table 3). Red or yellow classification was not significantly associated with the health outcomes of percent of births given at term, percent of births at satisfactory weight, or number of children testing positive for elevated blood lead levels (Table 4). Red and yellow classification was significantly associated with the teen birthrate (additional 23.61 (yellow) and 20.36 (red) births per 1000) (Table 4). The process outcome of percentage of births with prenatal care was not significant for red classification but was for yellow (5.06 percent fewer births) (Table 4). For CSA characteristics, red or yellow classification was associated with a significant increase of 0.87 (yellow) and 2.18 (red) additional liquor stores per 1000 residents but was not significantly associated with the rate of fast-food restaurants (Table 5).

**Table 4:** Multivariable ordinary least squares analysis of Baltimore HOLC and 2013 maternal and child health outcomes and processes.

HOLC Blue/Green ref	HOLC Yellow		HOLC Red		Median HH Income-$1000		African American-%	
Dependent variables: n = 54	Effect [95% CI]	P-value	Effect [95% CI]	P-value	Effect [95% CI]	P-value	Effect [95% CI]	P-value
teen pregnancy per 1000 female residents 15–19	23.61 [7.97, 39.26] *	**0.004**	20.36 [4.34, 36.37] *	**0.014**	-0.03 [-0.49, 0.43]	0.896	0.18 [-0.09, 0.45]	0.192
term birth-% of births	-1.49 [-3.79, 0.82]	0.201	-0.66 [-3.02, 1.7]	0.575	-0.04 [-0.11, 0.02]	0.198	-0.11 [-0.15, -0.07] *	**p<0.001**
satisfactory birth weight-% of births	-1.67 [-3.76, 0.41]	0.113	-0.89 [-3.03, 1.25]	0.406	-0.01 [-0.07, 0.05]	0.795	-0.09 [-0.13, -0.06] *	**p<0.001**
Received 1st trimester prenatal care-% of births	-5.06 [-8.7, -1.43] *	**0.007**	-1.82 [-5.54, 1.9]	0.331	0.19 [0.08, 0.3] *	**0.001**	-0.14 [-0.21, -0.08] *	**p<0.001**
age 0–6 elevated blood lead levels-%	0.67 [-0.67, 2]	0.32	0.19 [-1.15, 1.52]	0.78	0 [-0.04, 0.04]	0.987	0.01 [-0.01, 0.04]	0.215

* Significant at the 95% confidence level. Unit of analysis is the community statistical area. Dependent variables in rows, independent variables and covariates in columns. Mean and 95% CI reported. Maternal and child health variables are individually regressed on HOLC yellow and HOLC red vs. blue/green, with covariates of neighborhood median household income, proportion of African American residents. Teen pregnancy effects are reported as additional pregnancies per 1000 female residents for the relevant HOLC grade (yellow or red) as compared to the reference category of HOLC blue/green. All other effects are reported as additional percentage points for the relevant HOLC grade (yellow or red) as compared to the reference category of HOLC blue/green. Median household income and percent African American are controls and effects should not be interpreted independently.

**Table 5:** Multivariable ordinary least squares analysis of Baltimore HOLC and 2013 neighborhood characteristics.

HOLC Blue/Green ref	HOLC Yellow		HOLC Red		Median HH Income-$1000		African American-%	
Dependent variables: n = 54	Effect [95% CI]	P-value	Effect [95% CI]	P-value	Effect [95% CI]	P-value	Effect [95% CI]	P-value
liquor outlets-per 1000 residents	0.87 [0.09, 1.65] *	**0.03**	2.18 [1.38, 2.98] *	**p<0.001**	0.00 [-0.02, 0.02]	0.95	-0.01 [-0.03, 0.001]	0.08
fast food outlets-per 1000 residents	0.07 [-2.43, 2.57]	0.954	2.49 [-0.07, 5.05]	0.056	2.49 [-0.07, 5.05]	0.056	-0.03 [-0.07, 0.02]	0.229

* Significant at the 95% confidence level. Unit of analysis is the community statistical area. Dependent variables in columns, independent variables and covariates in rows. Mean and 95% CI reported. CSA neighborhood factors are individually regressed on HOLC yellow and HOLC red vs. blue/green, with covariates of neighborhood median household income, proportion of African American residents. Effects are reported as additional outlets per 1000 residents for the relevant HOLC grade (yellow or red) as compared to the reference category of HOLC blue/green. Median household income and percent African American are controls and effects should not be interpreted independently.

For our sensitivity analysis, 8 CSAs were excluded for having less than 25% HOLC coverage (CSAs 4, 9, 12, 30, 38, 44, 45, and 50). Five excluded CSAs were categorized as yellow, and the remaining 3 were distributed evenly among red, blue, and green. Rerunning our first model while excluding these CSAs resulted in effect sizes with the same directionality and significance, and the effect size was mildly stronger with yellow CSAs associated with a mean 5.78 years reduction (95% CI: 3.01, 7.69) and red CSAs associated with a mean 4.44 years reduction (95% CI: 1.61, 7.27).

## Discussion

### Summary

These results lend support to the current health impact of living in areas of Baltimore that were categorized as “hazardous” or “declining” by the HOLC in the 1930s, specifically with regards to life expectancy and age-differentiated mortality. To our knowledge, this is the first study to examine the association of redlining with age-differentiated mortality.

This study adds an additional health dimension of support to broader claims that past redlining still has impacts today. While Aaronson et al. [12] and Appel and Nickerson [21] focus on the current impact of redlining and yellowlining on residential segregation and household values, and Boone et al. [32] focus on the impact of redlining on greenspaces, this study lends support that there are associated ultimate impacts on life expectancy and mortality as well, at least in Baltimore. It also lends support to the hypothesis posed by Mendez et al. [31] that redlining has an impact on health outcomes. However, instead of using modern loan applications as a proxy for historical redlining [31], these results are based on the actual HOLC security maps. More broadly, these results provide support for the argument that neighborhood level characteristics [23,28–31] have an impact on health characteristics.

In addition to categorization, the HOLC maps themselves provide insight on the bias implicit in grade-assignment and how that historical social bias is associated with present day health effects. The descriptions in the HOLC maps note “detrimental influences” such as a “heavy concentration of foreigners” or characterising a preponderance of African American residents as an “infiltration”. At the time, HOLC grades suggested that whiter neighborhoods were “desirable” and colored neighborhoods “hazardous”. The gravity of these historical assignments is felt even today, as the HOLC maps were both a consequence of existing discriminatory lending practices and a cause of sustained disinvestment in neighborhoods.

#### Current associations of redlining with life expectancy

Even after controlling for racial composition and median household income, CSAs that were categorized as red or yellow had a 5-year shorter life expectancy than CSAs that had been categorized as green or blue. While the effect is small and we are not testing a full model for racial composition, racial composition as a control continues to have a significant association with life expectancy, which suggests that red/yellow categorization impact on life expectancy is not entirely due to the racial composition of CSAs. These results are consistent with Richardson et al. [40] which also found significant differences in life expectancy between the most and least redlined quartiles of census tracts nationwide.

Care must be taken in saying that the HOLC security maps by themselves caused the decline in life expectancy or increases in mortality at various age groups. Instead, as discussed by others [12,47], the HOLC security maps are likely a proxy for de jure and de facto government policy throughout the 20^th^ century that systematically disenfranchised and harmed the largely Black residents of those neighborhoods [9]. These include—but aren’t limited to—lending practices, restrictive racial covenants, and political choices for municipal investment not just in the 1930s but throughout the 20^th^ century and into today [47]. However, the initial state of racial discrimination in the policymaking of the 1930s enabled many of these other racist policies and still leaves a mark today. Aaronson et al. [12] found discontinuous city size-dependent differences in residential segregation by exploiting how HOLC maps were used in differently sized cities. This suggests that HOLC security maps may have been a driving force behind broader policies hostile to Black residents.

### Maternal and child health outcomes

O’Campo et al. [29] provide strong support for the notion that low birthweight was dependent on macro level neighborhood health factors at the census tract level in Baltimore. Krieger et al. [25] likewise provide evidence for New York City. However, HOLC categories in Baltimore are not associated with low birthweight or term birth. It is possible that HOLC categories at the CSA level are not sensitive enough to variations in birthweight by census tract, or that the sample size is too small to detect such variations. It is also possible that while historical HOLC categories are related to certain neighborhood characteristics (such as the significantly associated increased prevalence of liquor stores), they might not be related to current neighborhood characteristics that are relevant for a safe birthweight. We did find associations with increased teen pregnancy and reduced prenatal care in the first trimester. While we did not assess measures of the quality of maternal care, others have noted that redlining and residential segregation may reduce access to appropriate healthcare services [23,25,29].

While we found no association with infant mortality, Lynch et al. [27] noted that current investment patterns in census tracts interact with redlining and population displacement of Black residents by white residents that may drive infant mortality more than historical redlining patterns alone. This may suggest similar patterns for other maternal and child health outcomes. While analysis including the interaction of current investment patterns with redlining is beyond the scope of this paper, it deserves further study in the future.

### Age-differentiated increased mortality

The primary driver of increased mortality came from those in the 25–84 age range, especially in the 65–84 age range. There are multiple possible explanations, though the data do not capture cause of death. People living in redlined neighborhoods had less access to green spaces [32] and physical activity that might have an impact on the prevention and management of chronic conditions [6]. Multiple, lifelong encounters with hostile systems and racism can also lead to increased and premature mortality in adults [2,5].

### Liquor stores

CSAs categorized as red had more per capita liquor stores than those categorized as green or blue, while CSAs categorized as yellow had fewer. While our results for red categorized CSAs are congruent with findings from Trangenstein et al. [33], we found the opposite effect for yellow categorized CSAs. However, Trangenstein et al. used census tracts rather than CSAs.

## Limitations

The unit of analysis was the CSA, not the individual. As a result, there is a risk of ecological fallacy in these results and analysis. Rather, redlining at the neighborhood level may not be the same construct [49] as an individual’s exposure to redlining. Small sample size from using CSAs as the unit of analysis is a study limitation as well, and may especially be an issue with relatively rare outcomes such as elevated blood lead levels in any given year. CSAs are also larger than other potential geographic units of analysis like census tracts: a more geographically concentrated unit of analysis may offer better resolution and cleaner categorization of HOLC categories than CSAs could. Future studies could use restricted-access census tract level mortality data to reduce the impact of these limitations. Life expectancy data was calculated by Baltimore’s Health Department using 2013 mortality rates within each age category, and thus may not fully capture “true” life expectancy.

The study scope is limited to Baltimore, which reduces the generalizability of these results. Other redlining maps from across the US are available for use in a future study. However, Baltimore provided a convenience sample that included publicly available mortality and redlining data and has been central to the national conversation around redlining and residential segregation.

The main limitation is the passage of time. While this study adds to the literature that suggests that nearly century-old policies in Baltimore still have measurable and significant impacts on residents’ lives today, the time lag masks a great number of social changes that have taken place in the interim. These include the strong possibility of endogenous and path-dependent [20] changes such as gentrification and concomitant displacement of populations that were living in redlined neighborhoods in the 1930s. Certainly, the demographic makeup of Baltimore has changed drastically even in the last decade [50]. Additionally, the study does not account for the length of time residing in home/residence for Baltimore residents, and potential CSA or city out-migration; if there were mobility across CSAs in Baltimore, this would potentially serve as a mediator of the effect of redlining. These changes to the social and built environment may be either enhancing or blunting the impacts of policies set in the 1930s. We are unable to determine from this analysis whether the measured reduction in life expectancy in redlined CSAs is due to CSAs’ differing resource characteristics—as suggested by several other authors [12,21,28,29,32]—or simply changes in the demographic profile of a CSA through displacement and concentration.

## Policy implications

Further study to overcome these limitations is needed to draw more concrete policy lessons. However, potential policy implications exist given that the results of this study found disparate and significant differences in life expectancy. The primary policy implication is that discriminatory public or social policy—whether or not such a policy is intentionally discriminatory—in a realm such as housing can potentially have long-lasting, disparate, and large impacts on health. Additionally, contemporary African American activists and organizations in the 1930s presciently recognized the discriminatory impacts of how HOLC made its policy decisions [16]: members of marginalized and/or impacted communities should have control over every major policy-making process.

Since many of these issues are structural and historical, health interventions that do not focus on disparate health outcomes risk being “weighed down” by structural problems that predispose a population to worse health. If we take health equity and disparities research seriously, we should be examining the possibility of interventions that attempt to tackle some of these structural factors, including the possibility of reparations [7]. What Link and Phelan propose as “fundamental causes” [51] of health disparities—such as a lack of flexible resources of money, knowledge, social connections, and political power—may have even more fundamental causes rooted in power hierarchies such as racial, class, and gender subordination.

Like other studies of redlining, this study also may be relevant to ongoing policy disputes in Baltimore. Multiple current policy issues in Baltimore including mitigation policies against displacement, redevelopment in the city core of formerly redlined neighborhoods particularly by health services provisioners operating theoretically under the aegis of Maryland’s hospital Community Benefit policies, and the creation of a new police force to control access to those redeveloped areas, should be backdropped against these nearly century-long policies and their associations with current health.

## Future studies

Given these policy questions and some of the study limitations, there are multiple avenues for further study. A future study could use census tract-level mortality data and use census tracts or blocks instead of CSAs as the unit of analysis and could look nationwide beyond Baltimore. It could also use tract or block area data to look at demographic changes [50] to gauge whether or not a neighborhood’s community has been displaced.

Multiple authors [12,21,47] argue that continued diminished investment in neighborhoods due to redlining might be what leads to lower housing values and lower quality neighborhoods today. Gentrification then might be a countervailing trend that brings in resources that might ameliorate any potential neighborhood-level health impacts of redlining. Adding measures of gentrification over time might be a way to account for how neighborhood health status changes over time. However, gentrification also displaces populations from geographically delineated neighborhoods [50]. Redlining may also have current health and non-health impacts through intergenerational effects: housing and neighborhood quality impacts education and wealth accumulation [7,9] which in turn impacts the ability to provide for the next generation. In this case, while gentrification might change a neighborhood’s health status, it would not be due to improvements to infrastructure or wealth accumulation of the impacted community but rather by their replacement. Adding measures of community composition changes could be a way to account for these changes [50]. Additionally, adding analysis of current investment patterns [27] may help elucidate the ways that redlining interacted with population displacement.

Potential pathways from redlining through sustained disinvestment leading to exposure to hazards have been both theorized [9,23] and in some cases explored [27,28]. Future studies could look more closely at neighborhood factors like documented environmental hazards, exposure to policing, healthcare utilization, or mobility patterns to clarify potential causal pathways, including specifically in Baltimore.

Additional topics to explore on neighborhood quality could include the association of HOLC categories with hospital availability and quality, or the response times of EMS. Geographic data on police interactions could also be used to examine the relationship between HOLC categories and where communities are policed. Future studies could also more thoroughly examine childhood health outcomes and factors including asthma. Geocoded social data might also be an avenue for exploration: Chae et al. [52] found a strong geographical association between area racism as the proportion of Google searches containing the “N-word” in 196 designated market areas and Black mortality. Such an analysis could be extended to redlined neighborhoods.

## Conclusion

HOLC assigned categories for neighborhood quality in the 1930s are associated with present-day impacts on life expectancy, age-differentiated mortality, and other health outcomes.

## Supporting information

S1 File Regression output. (DO)
